# Monthly Summary of Med. Progress

**Published:** 1889-09

**Authors:** W. S. Wells


### MONTHLY SUMMARY OF MEDICAL PROGRESS.

By W. S. Wells, M. D.

Dr. A. Mueller, of Victoria, communicates to the *Australasian Med. Gazette* an article on the cure of poisonous snake-bites, by hypodermic injections of liq. strychniæ. He says:

"The diagnosis of snake-poisoning is not, in every case, as easy as it may appear to the uninitiated. No doubt, of course, remains where the snake has been seen to strike, or even bite and hang on to the bitten limb, and where the symptoms are fully developed. But frequently the snake is not seen to have bitten, but its proximity excites such terror as to simulate the symptoms of snake-poisoning. Terror, alone, has been known to cause death. The influence of terror and snake-poisoning are both capable of causing paralysis of the motor nerve centres."

Dr. Mueller calls attention to a fruitful source of error that has cost many human lives, viz.: That poisonous snakes, under all circumstances, leave only two punctures, and that in any alleged case of snake-bite, showing more than two punctures, we may safely infer and rest content that it was not inflicted by a poisonous snake. On the contrary, he has met with fatal cases in which the poisonous bite left one, two, three or four punctures, depending on the position of the reptile, as well as of the part bitten. When the bite has been received in a part capable of being ligatured, as the foot or hand, a boot-lace or string should be at once tightened as near the wound as possible, on the side toward the heart, to prevent absorption of the poison into the circulation.

With strychnine solution as the antidote, the difficulties in snake-bites are greatly lessened. Should a careful analysis of the history of a case not enable us to judge conclusively, whether the symptoms emanate from fear or real poisoning, a small injection of liq. strychniæ will, in case of mere fear, be quite sufficient to brace up the nervous system and restore confidence of recovery, with which all alarming symptoms dsappear. Their continuance would indicate the presence of snake-poisoning, and call for larger injections of the antidote. But if in one of these cases of false alarm, alcohol has been administered in excess, as is usually done, we find the patient in the condition of coma—alcoholic coma,—and we must diagnose this from snake-bite coma. In alcoholic coma, the pupil is as often contracted as dilated, and becomes greatly dilated in extreme cases only, but even then shows a sluggish reaction to light; the conjunctiva is invariably injected, the pulse slow and small, and the respiration slow, intermittent, and often stertorous. In snake-bite coma, the pupil is always greatly dilated and insensible to light, the conjunctiva pale and not injected, the pulse quick and small, and the respiration quick and shallow.

With regard to the use of alcohol in snake-bite, Dr. Mueller affirms that it is perfectly useless. No matter how large a quantity of whiskey or brandy is taken, its stimulating action does not become manifest by as much as a flush of the cheeks, until the snake-poison has been effectually counteracted by strychnine.

Strychnine in snake-bite acts with unerring certainty and precision of a chemical test. Purely physiological in action, it neutralizes the effects of the snake-poison, and, if pushed beyond the amount needed to neutralize the snake-poison, would itself act as a poison. Its poisonous effects, on the other hand, could be combatted by injections of snake-poison, could the latter be at hand in an emergency of poisoning by strychnine.

The amount of strychnin solution injected varied in the several cases cited, from seventeen minims to twenty minims per dose, or from one-sixth to one-fifth of a grain, repeated, from time to time, as the snake-poison symptoms returned. In one case, only one-fifteenth of a grain of strychnine, in all, divided into four injections, was required to effect a cure, but in this case a ligature had once been applied, and a small quantity of poison had been absorbed. The amount of strychnine required can only be estimated by the nature and virulence of the bite, and whether ligature and excision have been well performed. If the latter have been neglected, and the snake is known to be particularly venomous, a camparatively large quantity of strychnine, in divided injections, will be required, before safety will be assured.—*Popular Science News.*

